# A Guide to the Diseases of Children

**Published:** 1885-08

**Authors:** 


**A Guide to the Diseases of Children.** By JAMES FREDERICK GOODHART, M. D., F. R. C. P.; Assistant Physician to Guy's Hospital; Physician to the Evelina Hospital for Sick Children. Revised and edited by LOUIS STARR, M. D., Clinical Professor Diseases of Children in the Hospital of the University of Pennsylvania. With formulæ. Philadelphia: P. Blackiston, Son & Co., 1012 Walnut street. 1885.

It is mainly from a clinical point of view that diseases of children can be rationally regarded as a special subject, and in the volume before us almost every page bears evidence that the author's views are based upon a rich clinical experience, and one cannot but recognize that he has seen what he is writing about. It is, therefore, an eminently practical work, and well adapted either for the student or practitioner. The reader cannot fail to be pleased with its systematic arrangement and clearness of expression. We would especially commend Dr. Goodhart's statement that "Children do not often require a very energetic treatment with drugs, and probably he will be the best practitioner who lets Nature make her cure. Proper feeding ranks first in all treatment in early life." The space devoted to diet of children in health and disease will, perhaps, be thought by some insufficient; but we think the careful attention to details will be appreciated by the student, who often starts into practice with such limited notions on the subject, that many a mother knows more of what is actually required than he does. The American editor's additions are appropriate, and increase the value of the work.

